# Cxxc Finger Protein 1 Positively Regulates GM-CSF-Derived Macrophage Phagocytosis Through Csf2rα-Mediated Signaling

**DOI:** 10.3389/fimmu.2018.01885

**Published:** 2018-08-14

**Authors:** Zhaoyuan Hui, Lina Zhou, Zhonghui Xue, Lingfeng Zhou, Yikai Luo, Feng Lin, Xia Liu, Shenghui Hong, Wei Li, Di Wang, Linrong Lu, Jianli Wang, Lie Wang

**Affiliations:** ^1^Institute of Immunology, and Bone Marrow Transplantation Center, First Affiliated Hospital, Zhejiang University School of Medicine, Hangzhou, China; ^2^Institute of Hematology, Zhejiang University and Zhejiang Engineering Laboratory for Stem Cell and Immunotherapy, Hangzhou, China; ^3^Division of Hepatobiliary and Pancreatic Surgery, Department of Surgery, First Affiliated Hospital, Zhejiang University School of Medicine, Hangzhou, China; ^4^Institute of Immunology, Zhejiang University School of Medicine, Hangzhou, China; ^5^State Key Laboratory for Diagnosis and Treatment of Infectious Diseases, Collaborative Innovation Center for Diagnosis and Treatment of Infectious Diseases, First Affiliated Hospital, College of Medicine, Zhejiang University, Hangzhou, China; ^6^Laboraty Animal Center, Zhejiang University, Hangzhou, China

**Keywords:** Cxxc finger protein 1, histone modification, macrophage, phagocytosis, Csf2rα

## Abstract

Macrophages have a defensive function against bacteria through phagocytosis and the secretion of cytokines. Histone modifications play an essential role in macrophage functions. Here, we report that Cxxc finger protein 1 (CFP1), a key component of the SETD1 histone methyltransferase complex, promoted the phagocytic and bactericidal activity of GM-CSF-derived macrophages. CFP1-deficient mice were more susceptible to bacterial infection due to the decreased expression of Csf2rα, a subunit of the GM-CSF receptor essential for inflammation and alveolar macrophage development, through the loss of H3K4 modifications in the promoter of the Csf2rα gene. In addition, the lung tissues of CFP1-deficient mice exhibited spontaneous inflammatory symptoms, including both the infiltration of inflammatory cells and the accumulation of surfactant phospholipids and proteins. Furthermore, we showed that Csf2rα and PU.1 can partially rescue the defects in phagocytosis and in the intracellular killing of bacteria. Collectively, our data highlight the importance of CFP1 in the phagocytic and bactericidal activity of macrophages.

## Introduction

Innate immunity constitutes the first line of host defense against bacteria and is mediated by phagocytes such as macrophages. As a major type of innate immune cell, macrophages can phagocytose bacteria and secrete both proinflammatory cytokines and antimicrobial mediators (1). During bacterial infection, phagocytes such as macrophages can clear bacteria through phagocytosis, which includes the recognition, uptake, and degradation of bacteria. Thus, phagocytosis has been recognized as a critical component of the innate immune response to bacteria (2).

Recent studies have suggested that histone modifications play essential roles in macrophage development and function (3, 4). Histone modifications are important regulators of chromatin function and recruit specific nuclear factors in order to promote different cellular functions. These modifications include the methylation, acetylation, phosphorylation, or ubiquitylation of different lysine (K), arginine (R), serine (S), and other amino acid residues in histones (5). Different modifications have been proposed to be associated with different transcriptional states (6). For instance, H3K4me3, H3K36me3, H3K79me2, and H3/H4ac are associated with transcriptional activation, whereas H3K9me2/3, H3K27me3, and H4K20me3 are linked with gene repression (7).

Epigenetic regulators participate in macrophage functions mainly through affecting chromatin remodeling of specific genes and regulating their expression. For example, histone deacetylase 11 (HDAC11) represses IL10 expression by decreasing acetylation at the distal segment of the IL10 promoter (8). In addition, histone deacetylase 9 (HDAC9) represses PPAR-γ1 expression by decreasing total H3 and H4 acetylation at the promoters in order to promote inflammatory cytokine production (9). In addition, deficiency of the histone demethylase Jumonji domain containing-3 (Jmjd3) leads to decreased expression of IRF4 through affecting H3K27me3 and to impaired alternatively activated macrophage polarization (10). In contrast, the histone methyltransferase mixed-lineage leukemia 4 (Mll4) promotes Pigp expression through H3K4 trimethylation at the PigP promoter, which then enhances lipopolysaccharide (LPS)-triggered intracellular signals (11). These studies suggest that histone modification enzymes are important for regulating cytokine secretion from macrophages. However, it is unclear how many of these histone modifications affect macrophage functions.

Cxxc finger protein 1 (CFP1), as an epigenetic regulator, is an important component of the SETD1 complex (12, 13). CFP1 binds to DNA *via* its Cxxc finger domain and its PHD domain and recruits SETD1 to the promoters of actively transcribed CGI-associated genes (14, 15). CFP1-dependent H3K4me3 regulates H3K9 acetylation and histone acetyl transferase recruitment (16). Recent studies demonstrated that CFP1 plays a critical role in thymocyte development, and loss of CFP1 leads to a severe blockade of intrathymic T cell development (17). However, the role of CFP1-mediated H3K4me3 in macrophages remains unclear.

To address these questions, we used CFP1 conditional knockout mice. CFP1-deficient inflammatory macrophages exhibited impaired phagocytosis and intracellular killing of bacteria, which led to susceptibility to bacteria *in vivo*. Using RNA-seq analysis, we identified downregulated genes related to endocytosis and phagosome maturation in CFP1-deficient macrophages. We demonstrated that CFP1 could promote Csf2rα expression through mediating H3K4 trimethylation at the Csf2rα promoter. Additionally, CFP1-deficient macrophages exhibited similar morphology to GM-CSF-deficient alveolar macrophages (AMs). Furthermore, Csf2rα and its downstream protein PU.1 could rescue defects in CFP1-deficient macrophages. Here, we elucidated a crucial role for CFP1 in the positive regulation of phagocytosis and bactericidal activity.

## Materials and Methods

### Animals

The *Cxxc1*^fl/fl^ mouse strain has been described previously (17). LysM-Cre mice (004781) were originally from the Jackson Laboratory. All mice had a C57BL/6J genetic background. Mice of 8–12 weeks old were used for the *in vivo* animal experiments. These mouse experiments were approved by the Institutional Animal Care and Use Committee and were in strict accordance with good animal practice as defined by the Zhejiang University Laboratory Animal Center.

### Cell Preparation and Culture

We prepared and cultured bone marrow-derived macrophages as previously described (18). Briefly, macrophages were generated in complete RPMI-1640 medium containing 10 ng ml^−1^ recombinant mouse GM-CSF (PeproTech, NJ, USA), 10% FBS (HyClone, UT, USA), 2 mM glutamine, and 100 U/ml penicillin–streptomycin for 6 days with replacement of the medium every 2 days. Approximately 99% of the cells were F4/80^+^CD11b^+^ when analyzed by flow cytometry.

### Flow Cytometry and Antibodies

The following SunGene antibodies were used in our experiments: F4/80 (BM8, 1 µg ml^−1^), Ly6C (monst1, 1 µg ml^−1^), and MHCII (M5/114.15.2, 1 µg ml^−1^). The CD11b (M1/70, 1 µg ml^−1^), CD11c (N418, 1 µg ml^−1^), CD80 (16–10 A1, 1 µg ml^−1^), and CD86 (GL1, 1 µg ml^−1^) antibodies were from eBiosciences.

Single cells obtained from bone marrow, spleen, or macrophages were stained for 30 min at 4°C with the appropriate fluorophore-conjugated antibodies, washed, and resuspended with flow cytometry staining buffer (1% BSA in PBS). Stained cells were analyzed with a BD Calibur flow cytometer (BD Biosciences). For each experimental condition, 100,000 cells were acquired for analysis.

### Establishment of the Mouse Model for Bacterial Infection

Cxxc finger protein 1-deficient and wild-type mice were injected intravenously with 2 × 10^5^ or 5 × 10^5^ *Listeria monocytogenes* (*L. monocytogenes*) (10403S) or were injected intraperitoneally with 7 × 10^7^ *Escherichia coli* (*E. coli*) strain O111:B4. Then, spleens or livers were collected and weighed. Half of spleens or livers were weighed and lysed for measurement of colony-forming units (CFU) (*L. monocytogenes*). The CFU of whole spleens or livers were calculated according the percentage of weight in whole tissues. Serum was collected for the measurement of CFU of *E. coli*.

### Phagocytosis and Bacterial Killing Assay

Phagocytosis was assessed by flow cytometry. To assess phagocytosis, 5 × 10^5^ macrophages were reseeded and cultured for overnight. Then, heat-killed *L. monocytogenes* or *E. coli* labeled with Alexa Fluor 647 (Invitrogen) were added at a multiplicity of infection (MOI) of 10 or 50, and the macrophages were harvested immediately or were incubated for 1 h at 37°C. Then, the cells were extensively washed five times with cold PBS. The uptake of bacteria was assessed *via* flow cytometry; the data are expressed as the percentage of positive cells.

The bacterial killing assay was performed according to the protocol described in previous studies (19). Briefly, *L. monocytogenes* (MOI, 1) or *E. coli* (MOI, 20) were added to cells cultured in antibiotic-free medium. After 30 min, gentamicin (50 µg ml^−1^) was added, and the cells were incubated for another 30 min. The gentamicin was then removed by thoroughly washing the cells with PBS, and the cells were incubated in fresh medium until the appropriate time points. At each time point, the cells were lysed by the addition of distilled H_2_O, and the diluted aliquots were spread on brain–heart infusion agar plates (*L. monocytogenes*) or LB agar plates (*E. coli*). The number of CFU on the plates was counted after incubation overnight at 37°C.

### Liver and Spleen Histology

The large lobe of the liver and the entire spleen were removed from the mice 2 days post-infection (*L. monocytogenes*), rinsed in PBS, and fixed in formalin. The liver and spleen were treated with sequentially increasing concentrations of ethanol for dehydration and were embedded in paraffin, cut into 5-µm sections, and then stained with hematoxylin and eosin (H&E). The brightfield images were taken by Olympus BX51 microscope and equipped with an Olympus DP70 digital camera. The number of microabscesses was counted by six images (one image per mice). The spleen cellular destruction was evaluated by quantifying relative intact white pulp of six images (one image per mice).

### Lung Histology

The lungs were removed, fixed in formalin, and processed for H&E and periodic acid-Schiff (PAS) staining.

### Immunofluorescence and Confocal Microscopy

Macrophages were fixed in prewarmed 4% paraformaldehyde for 10 min and permeabilized with 0.2% Triton X-100 for 5 min. After the samples were blocked with 5% BSA, the cells were incubated for 1 h with phalloidin (1:50 in PBS containing 5% BSA, Invitrogen). Nuclei were stained with DAPI (Beyotime Biotechnology). Five random fields per condition were imaged using a confocal microscope (LSM800, Zeiss). The mean pixel intensity of GFP was measured with Zeiss ZEN 2 lite software (20). The level of phagocytosis is expressed as the ratio of the total area of GFP dots to overall cell number.

### RNA-seq and Analysis

Total RNA was extracted from wild-type and CFP1-deficient bone marrow-derived macrophages with TRIzol reagent (Invitrogen). Library construction and sequencing were performed on a BGISEQ-500 platform by Wuhan Genomic Institution (www.genomics.org.cn, BGI, Shenzhen, China). All reads were mapped to the mm10 mouse genome, and the uniquely mapped reads were subjected to RNA-seq data analysis using the Hierarchical Indexing for Spliced Alignment of Transcripts (HISAT) system (21).

### Chromatin Immunoprecipitation (ChIP) Analysis

Chromatin immunoprecipitation assays were performed using the ChIP-IT kit (Active Motif, USA) according to the manufacturers’ instructions with modifications. Briefly, macrophages were fixed in 1% formaldehyde. The crosslinked chromatin was sonicated using a Bioruptor UCD-200 sonicator in a 4°C water bath in order to obtain DNA fragments sized between 100 and 500 bp. Chromatin from 2 × 10^6^ cells was used for each ChIP experiment. Antibodies against CFP1 (Abcam, 5 mg for each ChIP reaction), H3K4me3 (Active Motif, 2.5 mg for each ChIP reaction), H3K9ac, and H3K27ac (Abcam, 4 mg for each ChIP reaction) were used. The ChIP qPCR primers are listed in Table S1 in Supplementary Material.

### Bronchoalveolar Lavage (BAL) Fluid

Mice were sacrificed by administration of intraperitoneal sodium pentobarbital. BAL fluid was isolated by cannulation of the trachea with a catheter. The lungs were flushed with 3 × 600 μl PBS, and BAL cells were harvested by centrifugation for 5 min at 1,500 r.p.m.

### Retrovirus Transfection

Retrovirus was prepared in Plat-E cells. Plat-E cells were transfected with pMX-IRES-GFP plasmids containing the indicated genes, the medium was replaced with fresh medium after 10 h, and the retrovirus-containing supernatant was collected after an additional 72 h.

Bone marrow cells were isolated from CFP1-deficient or wild-type mice. These cells were transduced on two successive days with retroviral vectors expressing the indicated genes. After the second transduction, the cells were resuspended in macrophage growth medium [RPMI-1640 medium supplemented with 10% (vol/vol) FCS, 100 U/ml penicillin, 100 µg ml^−1^ streptomycin, and 10 ng ml^−1^ GM-CSF]. The cells were cultured for 6 days and were then analyzed. For the phagocytosis assay in reconstituted macrophages, AF-647-labeled *L. monocytogenes* was added at an MOI of 10, and the cells were incubated for 40 min. For the bacterial killing assay, *L. monocytogenes* was added into the cells at an MOI of 10.

### Statistical Analysis

The mean and SD of four independent experiments is shown, except where otherwise indicated, and comparisons were performed by a two-tailed unpaired Student’s *t*-test. Significance levels (*P* values) are presented on the figures.

## Results

### Defective Phagocytosis and Intracellular Killing of Bacteria in CFP1-Deficient GM-CSF-Derived Macrophages

Cxxc finger protein 1 is an important epigenetic regulator and is crucial for vertebrate development. The loss of CFP1 in mice results in peri-implantation lethality (22). In CFP1 conditional knockout mice (Mx1 Cre), ablation of CFP1 results in a nearly complete loss of lineage-committed progenitors and mature cells after the induction of Cre expression in adult animals (23). To investigate the role of CFP1 in inflammatory macrophages, we crossed *Cxxc1*-floxed mice (*Cxxc1^fl/fl^*) (17) with LysM-Cre knock-in mice to delete CFP1 in neutrophils, monocytes, and macrophages. We performed qPCR and western blot analyses to detect the deficiency of CFP1 in GM-CSF-cultured bone marrow-derived macrophages, which confirmed that CFP1 was successfully deleted in the CFP1-deficient mice (Figures S1A,B in the Supplementary Material).

First, we addressed the effect of CFP1 on the populations of monocytes and macrophages in CFP1-deficient mice. There was no significant difference between CFP1-deficient and wild-type mice in the percentage or cell number of F4/80^+^CD11b^+^ macrophages and Ly6C^hi^CD11b^+^ inflammatory monocytes in the bone marrow (Figure S1C in Supplementary Material) and spleen (Figure S1D in the Supplementary Material). Additionally, the expression of F4/80, CD11b, CD11c, and antigen presentation-related molecules such as MHCII, CD80, and CD86 were not affected in CFP1-deficient macrophages after *in vitro* culture with GM-CSF for 6 days (Figure S1E in the Supplementary Material).

To investigate the role of CFP1 in GM-CSF-derived macrophages, we examined the cytokine production of macrophages in response to lipoteichoic acid (LTA) and LPS. LTA is a major constituent of the cell wall of Gram-positive bacteria, and LPS is the major outer surface membrane component present in almost all Gram-negative bacteria. After stimulation with LTA or LPS, the mRNA level and protein level of IL6 and iNOS, compared to those of the wild-type macrophages, were not affected in CFP1-deficient macrophages, while IL12p40 decreased and TNF-α decreased only after stimulation with LPS (Figures S2A–C in the Supplementary Material).

**Figure 1:**
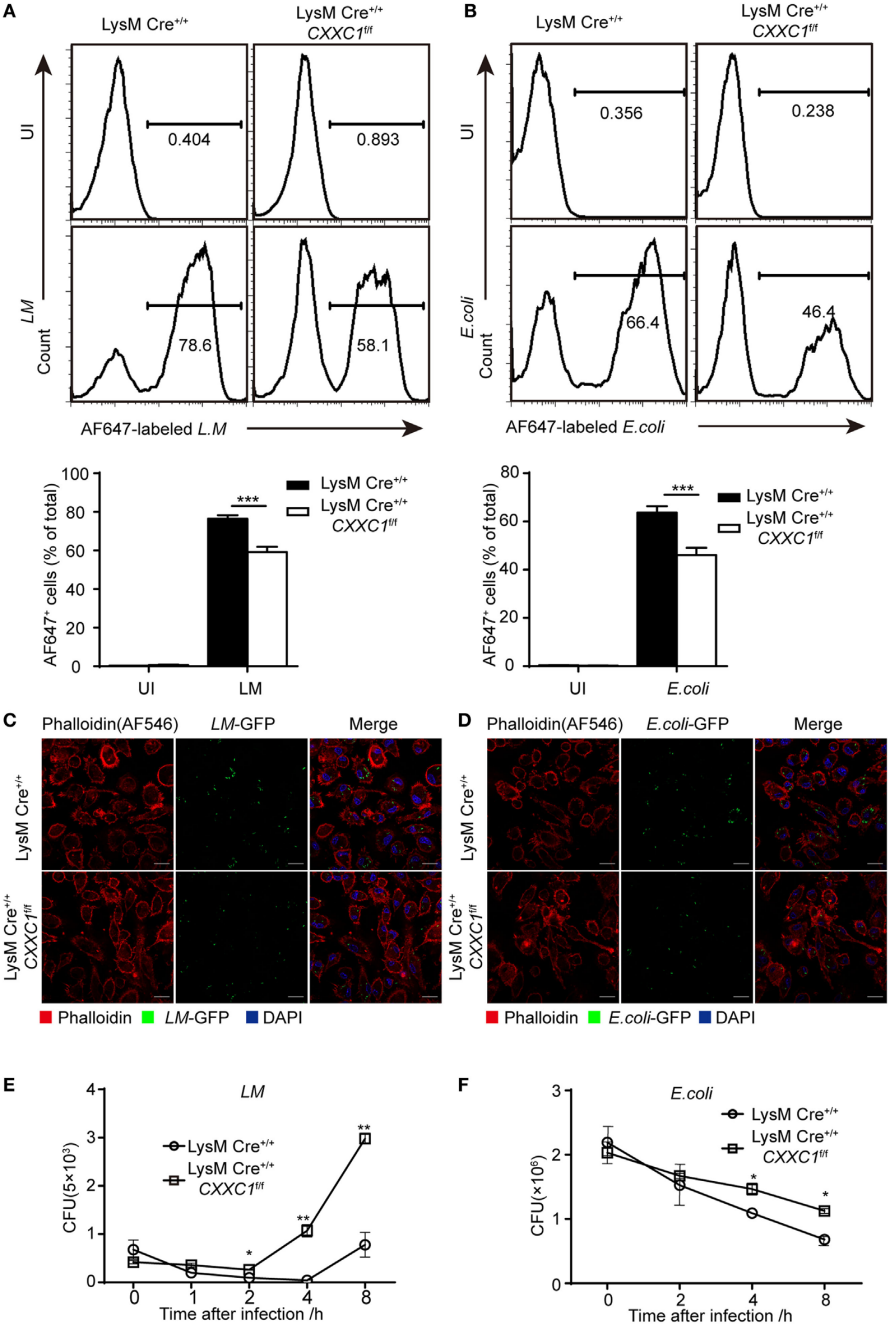
Compared with macrophages from wild-type mice, Cxxc finger protein 1 (CFP1)-deficient macrophages showed defective phagocytosis and intracellular killing of bacteria. **(A,B)** Flow cytometry of wild-type (black rectangle) and CFP1-deficient macrophages (white rectangle) left uninfected (UI) or infected for 60 min with either AF647-labeled *Listeria monocytogenes* (AF647-*LM*) at a multiplicity of infection (MOI) of 10 **(A)** or with AF647-labeled *E. coli* (AF647-*E. coli*) at an MOI of 20 **(B)**. **(C,D)** Confocal microscopy images of GM-CSF-derived CFP1-deficient macrophages and wild-type cells incubated with *L.M*-GFP (MOI, 10) **(C)** or *E. coli*-GFP (MOI, 20) **(D)**. The scale bars represent 20 µm. **(E,F)** Pathogen burden in wild-type (white circle) and CFP1-deficient (white square) macrophages infected with *L. monocytogenes* (MOI, 1) or *E. coli* (MOI, 20), presented as colony-forming units (CFU). The mean and SD of four independent experiments are shown. *P < 0.05, **P < 0.01 and ***P < 0.001 indicate significant differences between groups as determined by Student’s *t*-test.

Next, we focused on macrophage effector functions. *Listeria monocytogenes* (*L. monocytogenes*) is a Gram-positive intracellular bacterium (24). *Escherichia coli* (*E. coli*) is a Gram-negative extracellular bacterium. We assessed bacterial phagocytosis by incubating fluorescein isothiocyanate-labeled *L. monocytogenes* or *E. coli* with macrophages derived from CFP1-deficient mice and wild-type mice. Flow cytometry showed that CFP1-deficient macrophages exhibited less phagocytosis of both *L. monocytogenes* and *E. coli* than wild-type macrophages did (Figures 1A,B). Furthermore, we applied immunofluorescence microscopy to evaluate phagocytosis. Similar results were obtained by infecting macrophages with live *L. monocytogenes*-GFP and *E. coli*-GFP that could stably express green fluorescent protein (Figures 1C,D). However, the number of live intracellular bacteria was significantly higher in CFP1-deficient macrophages than in wild-type macrophages, as measured at late time points after bacterial infection (Figures 1E,F), which indicated that in addition to showing decreased uptake of bacteria, CFP1-deficient macrophages were significantly defective in the intracellular killing of bacteria.

These results indicated that CFP1 was required for the phagocytic and bactericidal activity of inflammatory macrophages and that these activities were important for efficiently clearing bacteria.

### CFP1-Deficient Mice Were More Susceptible to Bacteria

**Figure 2:**
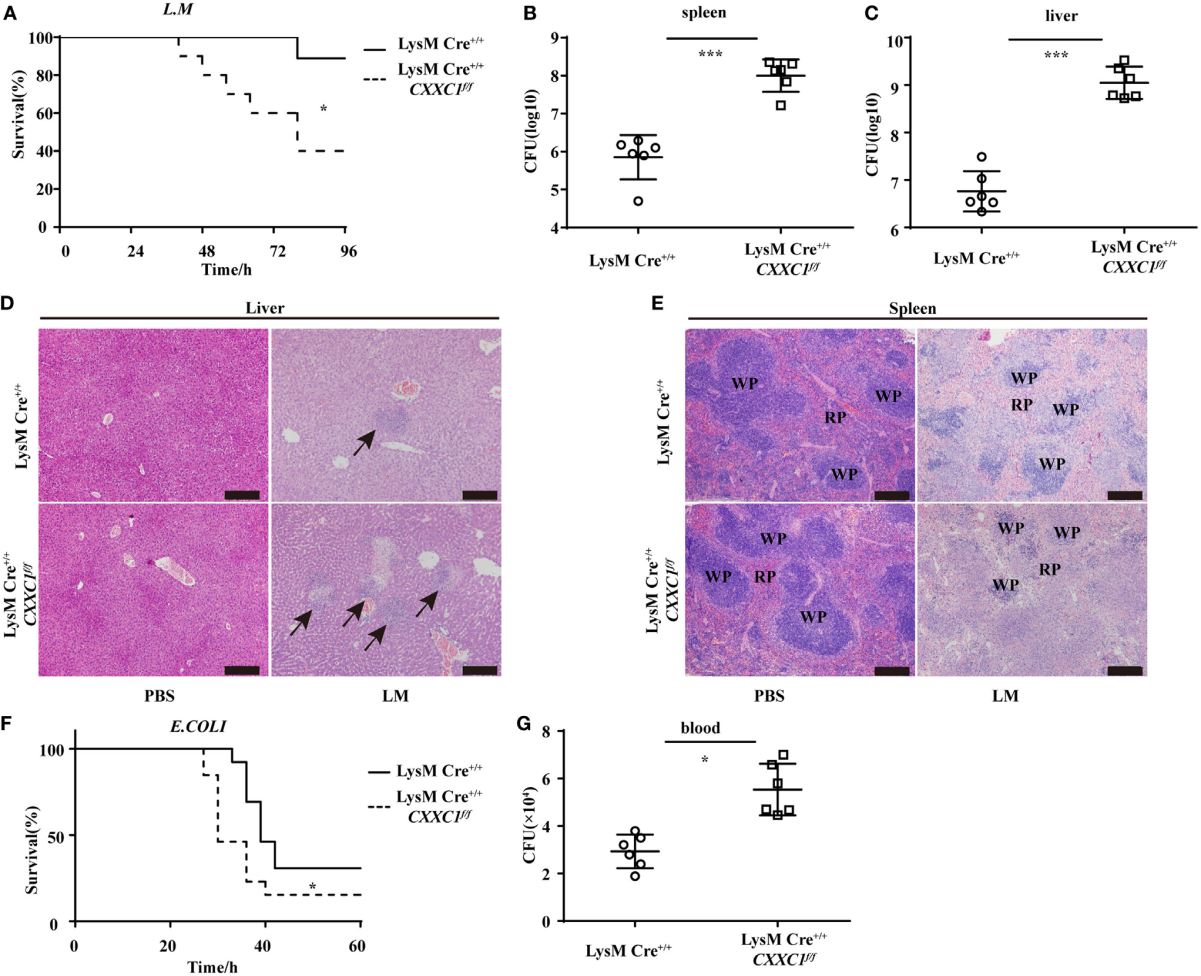
Cxxc finger protein 1 (CFP1)-deficient mice were more susceptible to *Listeria monocytogenes* and *E. coli* infection. **(A)** Survival of wild-type (*n* = 10) and CFP1-deficient (*n* = 11) mice injected in the tail vein with 5 × 10^5^ *L. monocytogenes* and monitored every hour after infection. **(B,C)** Bacterial load in the spleen **(B)** or liver **(C)** of wild-type (white circle) and CFP1-deficient (white square) mice injected in the tail vein with 1 × 10^5^ *L. monocytogenes* assessed on day 2 after infection (*n* = 6 mice per genotype). **(D,E)** Hematoxylin and eosin staining of liver **(D)** and spleen **(E)** tissue from wild-type and CFP1-deficient mice 48 h after tail vein injection of 1 × 10^5^ *L. monocytogenes*. The scale bars represent 200 µm. **(F)** Survival of wild-type and CFP1-deficient mice (*n* = 13) injected intraperitoneally with 3 × 10^8^ *E. coli* and monitored every hour after infection. **(G)** Bacterial load in blood (10 µl) collected 24 h of wild-type (white circle) and CFP1-deficient (white square) mice after infection as in **(F)**. CFU, colony-forming units (*n* = 6 mice per genotype). Each symbol represents an individual mouse; the small horizontal lines indicate the mean **(B,C,G)**. *P < 0.05 and ***P < 0.001 indicate significant differences between groups as determined by Student’s *t*-test.

To verify the role of CFP1 *in vivo*, we challenged CFP1-deficient mice with *L. monocytogenes* or *E. coli*. In response to intravenous infection with *L. monocytogenes*, we found that approximately 40% of the CFP1-deficient mice survived for 96 h, whereas approximately 90% of the wild-type mice survived for this time period (Figure 2A). We also assessed the response of wild-type and CFP1-deficient mice to intravenous infection with *L. monocytogenes*, measured as total CFU in the liver and spleen at day 2 after infection. CFP1-deficient mice had significantly more bacteria in the liver and spleen than did wild-type mice (Figures 2B,C). To evaluate the microabscess formation in the liver of *L. monocytogenes*-infected wild-type and CFP1-deficient mice, we stained the liver with H&E at 48 h post infection and found that there were more microabscesses in the liver of CFP1-deficient mice than in the liver of wild-type mice (Figure 2D; Figure S3A in the Supplementary Material). We also examined the spleen histology of infected wild-type and CFP1-deficient mice by H&E staining. The cellular destruction observed in the white pulp of the spleen from CFP1-deficient mice was substantially more severe than that observed in the wild-type mice (Figure 2E; Figures S3A in Supplementary Material).

In response to intraperitoneal infection with *E. coli*, 50% of the CFP1-deficient mice were dead at 30 h after infection, while all wild-type mice were alive at that time (Figure 2F). Additionally, the CFP1-deficient mice had a higher bacterial load in the blood than did the wild-type mice (Figure 2G).

These data suggested that the CFP1-deficient mice were more susceptible to *L. monocytogenes* and *E. coli* infection than were wild-type mice, which was in accordance with the *in vitro* results.

### RNA-seq Showed Impaired Expression of Phagosome Maturation-Related Genes in CFP1-Deficient Macrophages

**Figure 3:**
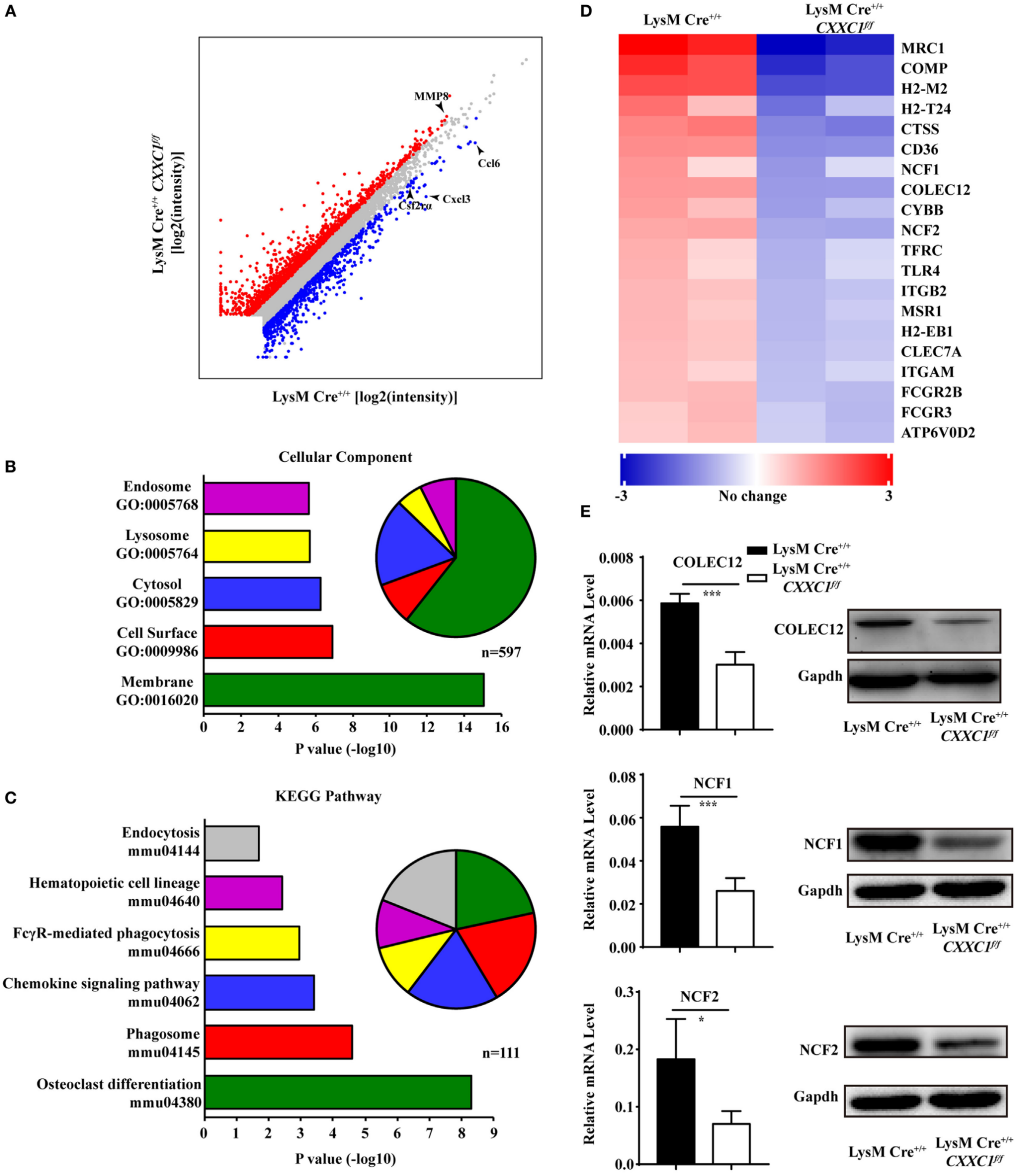
Cxxc finger protein 1 (CFP1)-deficient macrophages showed decreased expression of phagosome pathway-related genes. **(A)** Scatter plot showing gene expression changes in wild-type and CFP1-deficient macrophages. Downregulated genes are indicated in blue; upregulated genes, in red. **(B)** GO analysis of the downregulated genes. Left: enriched GO categories (*P* < 0.05). Right: pie chart showing the number of genes in each GO category. **(C)** KEGG analysis of the downregulated genes. Left: enriched GO categories (*P* < 0.05). Right: pie chart showing the number of genes in each GO category. **(D)** KEGG pathway analysis of genes in phagosome-related pathways, presented as the expression in CFP1-deficient macrophages relative to that in wild-type macrophages. **(E)** The mRNA and protein level of COLEC12, NCF1, and NCF2 in wild-type (black rectangle) and CFP1-deficient (white rectangle) macrophages. The mean and SD of four independent experiments are shown. *P < 0.05 and ***P < 0.001 indicate significant differences between groups as determined by Student’s *t*-test.

To uncover CFP1-dependent genes relevant to phagocytic and bactericidal activity in macrophages, we compared the transcriptomes of wild-type and CFP1-deficient macrophages by high-throughput sequencing of cDNA libraries (RNA-seq). Considering a fold change of 1.5 and a *P* < 0.05 to be the threshold parameters, we found that 792 genes, including Ccl6, Cxcl3, and Csf2rα, were downregulated by CFP1 knockdown in CFP1-deficient cells. However, 1,341 genes, for example, MMP8, were upregulated (Figure 3A).

Published work has reported that CFP1 was required for gene expression through H3K4 trimethylation and H3K9 acetylation (16), and the loss of CFP1 led to the increased expression of neighboring genes through aberrant H3K4me3 accumulation at regulatory regions (13). In our study, few of the upregulated genes were associated with macrophages phagocytosis, which may explain the inappropriate expression due to the loss of CFP1. Thus, we focused on downregulated genes. A gene ontology cellular component analysis of the downregulated genes using DAVID (25) revealed that many genes were associated with endosomes and lysosomes (Figure 3B). Of the downregulated genes, 44 were identified to be associated with the endosome GO term, and 32 were found to be associated with the lysosome GO term. Furthermore, we also performed a KEGG pathway analysis of the downregulated genes and found highly significant enrichment of many genes associated with a variety of phagosome, hematopoietic cell lineage, and endocytosis pathways (Figure 3C). 20 of those enriched genes, including COLEC12, NCF1, and NCF2, were associated with the KEGG phagosome pathway (Figure 3D). COLEC12 plays an important role in the uptake of bacteria in both CHO-K1 cells (26, 27) and human vascular endothelial cells (28), while NCF1 and NCF2 are associated with bactericidal activity in phagocytes (29). We measured the expression of those genes and obtained the same results as we obtained by the RNA-seq analysis (Figure 3E). These findings further support the conclusion that CFP1 contributes to phagocytic and bactericidal activity.

### CFP1-Mediated H3K4 Trimethylation at the Csf2rα Promoter and Promoted Csf2rα Expression

**Figure 4:**
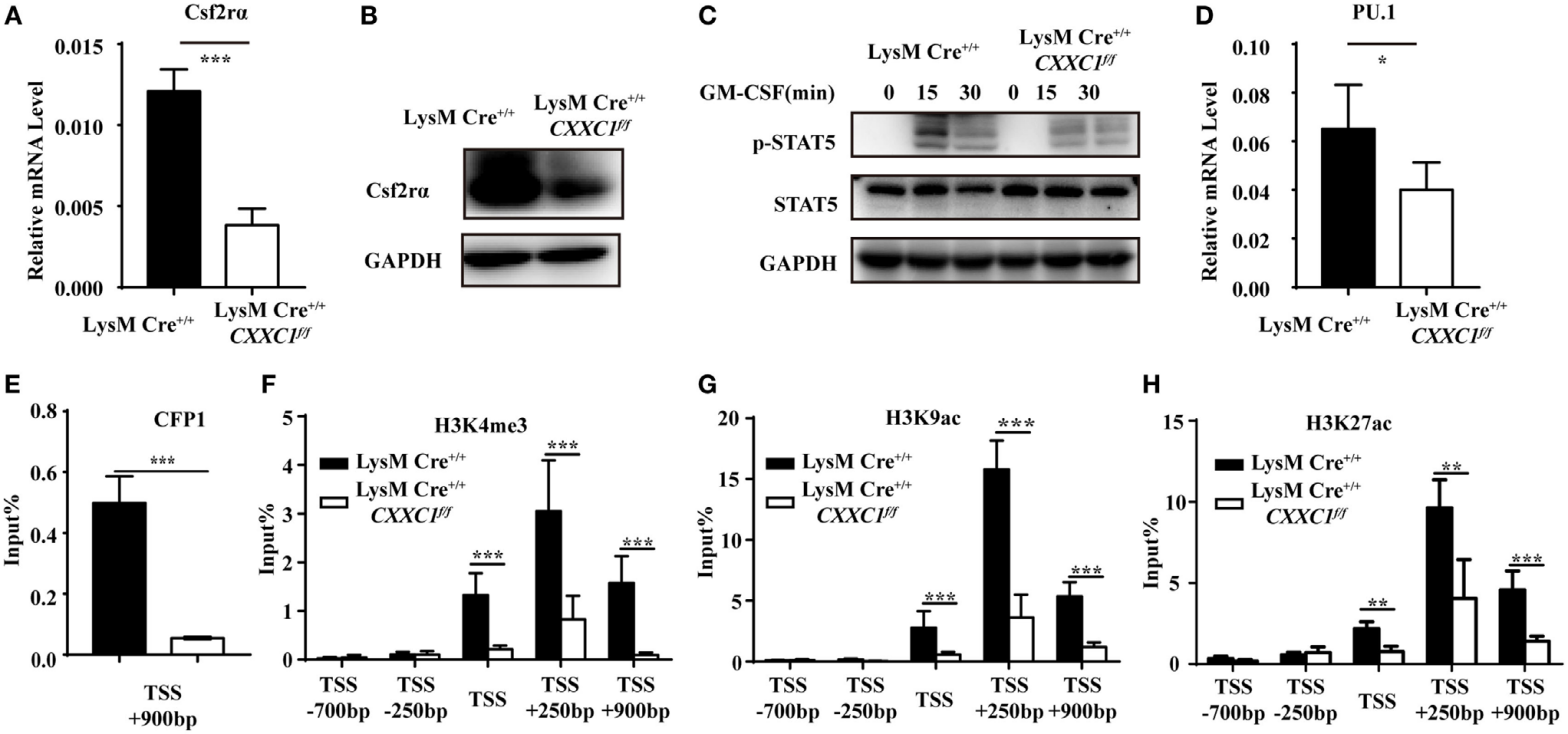
Cxxc finger protein 1 (CFP1) bound to the Csf2rα promoter region and was associated with H3K4me3, H3K9ac, and H3K27ac. **(A,B)** The expression of Csf2rα at the mRNA **(A)** and protein **(B)** level in wild-type (black rectangle) and CFP1-deficient (white rectangle) macrophages. **(C)** Immunoblot analysis of phosphorylated (p-) STAT5 and total STAT5 and GAPDH in wild-type and CFP1-deficient macrophages stimulated for 0–30 min (upper lanes) with GM-CSF (10 ng ml^−1^). **(D)** The expression of PU.1 at the mRNA level in wild-type (black rectangle) and CFP1-deficient (white rectangle) macrophages. **(E–H)** Chromatin immunoprecipitation analysis of endogenous CFP1 **(E)**, H3K4me3 **(F)**, H3K9ac **(G)**, H3K9ac **(H)** in wild-type (black rectangle) and CFP1-deficient (white rectangle) macrophages, followed by real-time PCR analysis for specific enrichment of each modification at the Csf2rα promoter. The primer positions are relative to the TSS of Csf2rα. The mean and SD of four independent experiments are shown. *P < 0.05, **P < 0.01 and ***P < 0.001 indicate significant differences between groups as determined by Student’s *t*-test.

To determine the mechanistic basis for the impaired phagocytosis observed in CFP1-deficient macrophages, we inspected the genes highly expressed in wild-type macrophages but downregulated as a result of the absence of CFP1. These genes should possess long CpG islands, since genome-wide ChIP sequencing shows a tight association between CFP1 and H3K4me3 at CGIs (14). We found that Csf2rα was at the top of the list of candidate genes; Csf2rα, which possesses a long CGI (2,821 bp), not only was highly expressed in macrophages and but was also significantly decreased in CFP1-deficient macrophages. Csf2rα is known to be a subunit of the GM-CSF receptor and can bind to GM-CSF to activate the GM-CSF signaling pathway mediated by JAK2 and STAT5 phosphorylation, which is critical for governing both differentiation and the inflammatory signature (30, 31). We found that the expression of Csf2rα at both the mRNA and protein levels dramatically decreased in CFP1-deficient macrophages compared with wild-type macrophages (Figures 4A,B). Additionally, compared with that in wild-type cells, STAT5 phosphorylation was decreased in CFP1-deficient macrophages after stimulation with GM-CSF (Figure 4C). Abundant evidence verified that GM-CSF promotes PU.1 expression in AMs and myeloid cells and that constitutive PU.1 expression in GM-CSF-deficient AMs could rescue all the phenotypic abnormalities (32, 33). In our study, PU.1 expression was reduced in CFP1-deficient macrophages compared with that in wild-type macrophages, as expected (Figure 4D).

Cxxc finger protein 1 has been shown to recruit the Setd1A complex in order to promote deposition of H3K4me3. We, therefore, hypothesized that CFP1 would promote Csf2rα expression by introducing activating marks at the Csf2rα promoter region. To determine whether CFP1 can bind to the Csf2rα promoter or not, we used a chromatin immunoprecipitation (ChIP) assay with a CFP1-specific antibody. In the ChIP assay, we found less enrichment in the binding of CGI at the Csf2rα promoter in CFP1-deficient macrophages compared to this binding in wild-type cells (Figure 4E). This result indicated that CFP1 bound to the Csf2rα promoter. To address whether CFP1 binding exerted an influence on altering chromatin modification, we performed ChIP analysis of H3K4me3 in wild-type and CFP1-deficient macrophages. We found that CFP1-deficient macrophages had less H3K4me3 at the promoter regions than wild-type cells (Figure 4F). In addition, the enrichment of the activating marks H3K9ac and H3K27ac was significantly decreased in CFP1-deficient macrophages compared with that in wild-type macrophages (Figures 4G,H). Considering all these data, we concluded that CFP1 mediated the promotion of Csf2rα expression at the chromatin level.

### CFP1-Deficient Macrophages and Mice Exhibited an Abnormal Phenotype

**Figure 5:**
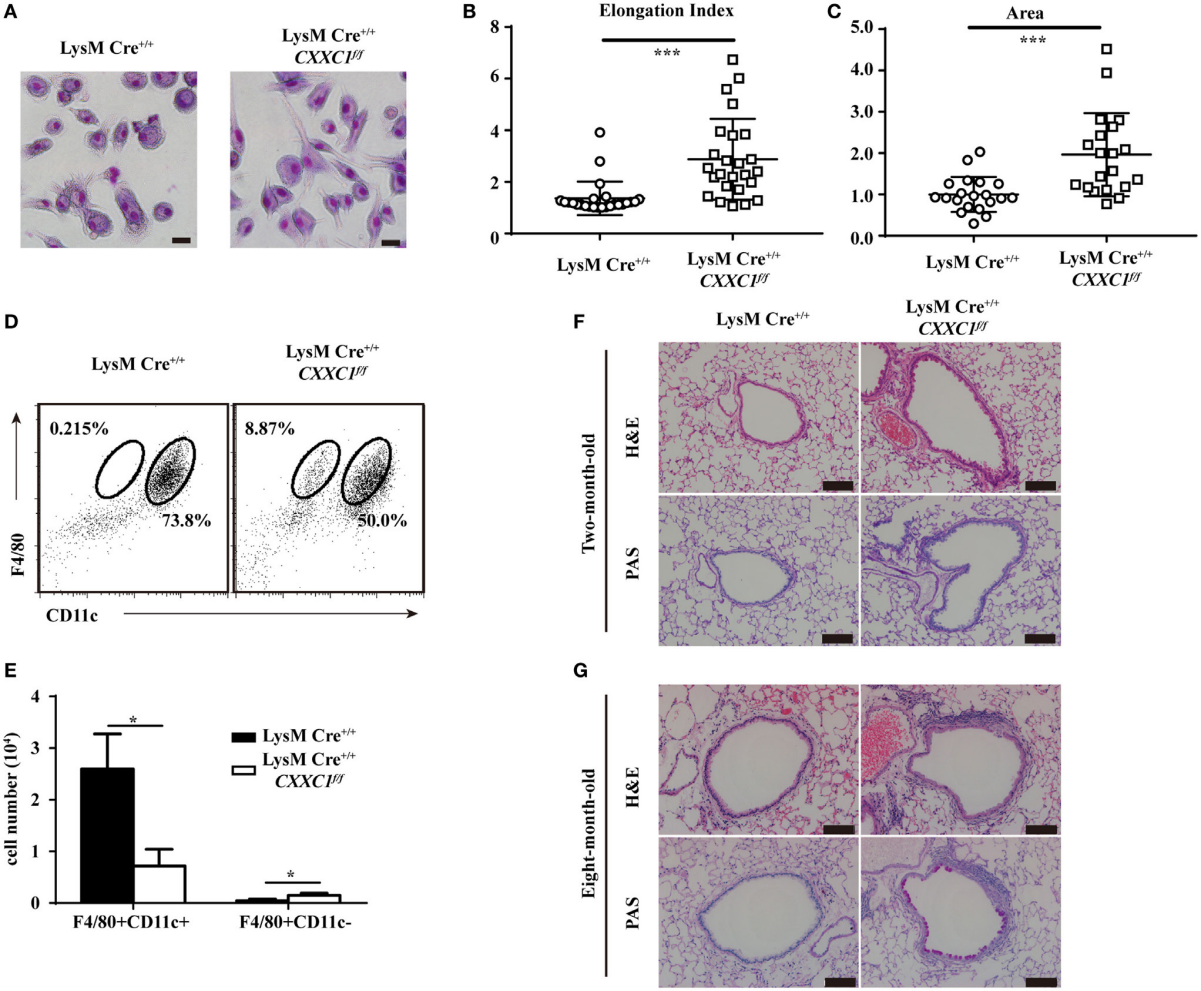
Cxxc finger protein 1 (CFP1)-deficient macrophages and mouse lung showed abnormal phenotypes. **(A)** Photomicrographs of wild-type and CFP1-deficient macrophages stained with May–Grünwald and Giemsa, scale bar: 100 µm. **(B)** Quantification of elongation, measured as the length of the long axis divided by the length of the short axis, for wild-type (white circle) and CFP1-deficient macrophages (white square). **(C)** Quantification of area for wild-type (white circle) and CFP1-deficient (white square) macrophages. The normalized area of the wild-type macrophages was arbitrarily set as 1. **(D,E)** Identification of alveolar macrophages (F4/80^+^CD11c^hi^) in the bronchoalveolar lavage fluid of wild-type (black rectangle) and CFP1-deficient (white rectangle) mice. **(F,G)** Panels show hematoxylin and eosin- and periodic acid-Schiff-stained histological lung sections of 2-month-old **(F)** and 8-month-old **(G)** wild-type and CFP1-deficient mice. Scale bars represent 100 µm. Each symbol represents an individual cell; the small horizontal lines indicate the mean **(B,C)**. The mean and SD of four independent experiments are shown. *P < 0.05 and ***P < 0.001 indicate significant differences between groups as determined by Student’s *t*-test.

GM-CSF signaling has been demonstrated to be essential for AM differentiation and maturation as well as for the regulation of surfactant homeostasis and host lung defense (31, 34, 35). An increasing number of studies have shown that cultured GM-CSF-deficient AMs were large and fat (33) and that they displayed impaired phagocytic and bactericidal activity. Moreover, GM-CSF- and CSF2rβ-deficient mice both exhibited an absence of AMs in BAL fluid and pulmonary alveolar proteinosis (36, 37). In our study, CFP1-deficient macrophages exhibited an atypical elongated and large morphology, whereas wild-type macrophages presented the typical pancake-like shape of inflammatory macrophages (Figures 5A–C). The number of F4/80^+^CD11c^+^ AMs decreased substantially in the BAL fluid of 8-week-old CFP1-deficient mice compared to that in the BAL fluid of wild-type mice, while the number of abnormal F4/80^+^CD11c^−^ cells increased substantially, and this effect was not observed in wild-type mice (Figures 5D,E). Histopathological examination of stained lung tissue showed increased inflammatory cell infiltration in 2-month-old CFP1-deficient mice compared with that in wild-type mice, and PAS staining exhibited normal results in CFP1-deficient mice and in wild-type mice (Figure 5F). However, compared with wild-type mice, 8-month-old CFP1-deficient mice showed both an infiltration of inflammatory cells and an accumulation of surfactant phospholipids and proteins in the lung tissue (Figure 5G).

Taken together, these data suggested that CFP1-deficient macrophages exhibited an atypical elongated and large morphology and that CFP1-deficient mice were susceptible to suffering from spontaneous inflammatory symptoms in the lungs.

### Restoring Csf2rα and PU.1 Expression Partially Rescued Phagocytic and Bactericidal Activity in CFP1-Deficient Macrophages

**Figure 6:**
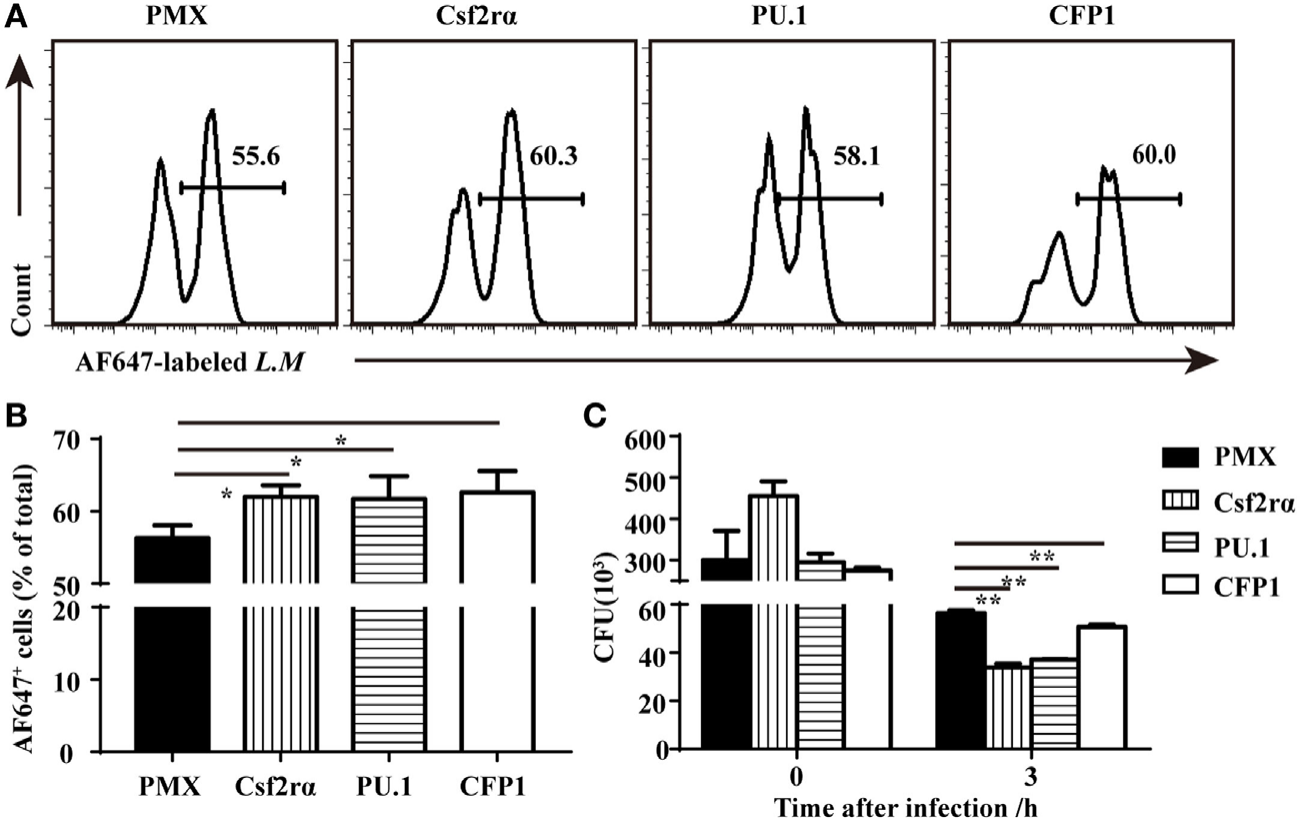
Csf2rα partially reversed the defects in the phagocytic and bactericidal activity of Cxxc finger protein 1 (CFP1)-deficient macrophages. **(A,B)** Flow cytometry analysis of CFP1-deficient (black rectangle) and Csf2rα- (vertical stripe rectangle), PU.1- (horizontal stripe rectangle), and CFP1-reconstituted (white rectangle) macrophages infected for 40 min with AF647-labeled *L. monocytogenes* (AF647-LM) at a multiplicity of infection (MOI) of 5. **(C)** Pathogen burden in CFP1-deficient (black rectangle) and Csf2rα- (vertical stripe rectangle), PU.1- (horizontal stripe rectangle), and CFP1-reconstituted (white rectangle) macrophages infected with *L. monocytogenes* (MOI, 10), presented as colony-forming units (CFU). The mean and SD of four independent experiments are shown. *P < 0.05 and **P < 0.01 indicate significant differences between groups as determined by Student’s *t*-test.

To determine if the re-expression of Csf2rα and PU.1 in CFP1-deficient macrophages was sufficient to rescue the defects in phagocytic and bactericidal activity, we infected CFP1-deficient macrophages with retroviruses expressing CFP1, Csf2rα, or PU.1 cDNA, and the transfection efficiency was about 30–40%. The phagocytosis of *L.M* was increased in the CFP1-reconstituted cells, which were used as a positive control (Figures 6A,B). Flow cytometry analysis also showed that the re-expression of Csf2rα and PU.1 in CFP1-deficient macrophages partially restored the phagocytosis of *L.M* (Figures 6A,B). Later, we evaluated whether the intracellular killing of *L.M* in CFP1-deficient macrophages could be rescued by the re-expression of CFP1, Csf2rα, or PU.1. The results showed that the cells reconstituted with CFP1 demonstrated an increased killing ability against *L.M* compared with that of CFP1-deficient macrophages (Figure 6C). In addition, the number of live intracellular *L.M* was dramatically decreased in the Csf2rα- and PU.1-reconstituted cells at late time points after infection (Figure 6C). These data demonstrated that Csf2rα and PU.1 could partially rescue the phagocytic and bactericidal activity of CFP1-deficient macrophages.

## Discussion

Epigenetic modifications such as histone modifications are becoming recognized as being far more important than originally thought in macrophage development and function. Many histone methyltransferases have been identified as playing an indispensable role in macrophage functions (3, 4). For example, loss of Mll4 leads to hypo-responsiveness to LPS (11). In addition, Ash1l-silenced macrophages demonstrate increased production of proinflammatory cytokines (38). Here, we illustrated the role of CFP1 in phagocytosis, and our results shed new light on the epigenetic regulation of macrophage effector functions.

As an epigenetic regulator, CFP1 guides H3K4me3 deposition by the SETD1 complex and is essential for the expression of target genes, which play important roles in hematopoiesis (23), thymocyte development (17), and oocyte development (39). In our study, we found few defects in macrophage development and cytokine production in CFP1-deficient mice.

The efficient clearance of bacteria depends on phagocytosis by macrophages. Our studies demonstrated that CFP1 plays a crucial role in promoting the phagocytic and bactericidal activity of GM-CSF-derived macrophage. Heretofore, macrophage-related studies of histone modifications focused on cytokine production. Only a few reports showed the role of histone modifications in macrophage phagocytosis. For example, the histone methyltransferase mixed-lineage leukemia 1 repressed the phagocytic and bactericidal activity of macrophages *in vitro* (40). In contrast, our results showed that CFP1 promoted the phagocytic and bactericidal activity of macrophages, and we verified this result *in vivo*.

In the KEGG analysis of the downregulated genes identified by RNA-seq, we also found that a high enrichment of genes associated with FcγR-mediated phagocytosis, a result that was in accordance with the impaired phagocytic function of GM-CSF-deficient AMs (41). Among the downregulated genes, we found some genes associated with phagocytosis, such as COLEC12, NCF1, and NCF2.

Furthermore, we identified Csf2rα as a target gene of CFP1 and showed that CFP1 promoted the expression of Csf2rα through a histone modification-dependent effect. Indeed, CFP1-deficient macrophages displayed an atypical elongated and large morphology similar to that of GM-CSF-deficient AMs. Moreover, CFP1-deficient mice exhibited spontaneous inflammatory symptoms in the lungs that resembled the phenotype of GM-CSF-deficient mice. These data suggested that CFP1 maintained the ability for phagocytosis and the intracellular killing of bacteria through promoting Csf2rα expression in GM-CSF-derived macrophages. In addition, we found that Csf2rα and PU.1 overexpression in CFP1-deficient cells reversed the decrease in phagocytic and bactericidal activity.

In our study, no proinflammatory cytokines were affected except for IL12-p40 in CFP1-deficient macrophages after stimulation with LTA or LPS. This result is supported by the prior observation that the production of IL12 was severely attenuated in AMs from GM-CSF deficient mice (41). A previous study showed that mutations in Csf2rα lead to pulmonary alveolar proteinosis in humans (42). However, the lungs of CFP1-deficient mice exhibited only slight chronic inflammation-related autoimmune symptoms, as well as an increased infiltration of inflammatory cells and an accumulation of phospholipids and proteins. This effect may have occurred because Csf2rα expression was decreased in CFP1-deficient macrophages but not completely absent. Although we found that Csf2rα was the main target of CFP1, we could still not exclude other target genes.

Collectively, we have demonstrated that CFP1 plays a crucial role in GM-CSF-derived macrophages, in which it positively regulates phagocytic and bactericidal activity mainly through promoting Csf2rα expression and the subsequent activation of downstream pathways. CFP1 can accumulate at CGI in the Csf2rα promoter, enhance H3K4 trimethylation, and thus promote Csf2rα expression. Thus, CFP1 acts as a promoter of macrophage phagocytosis and the intracellular killing of bacteria in a manner dependent on histone modification. More importantly, our findings provide the insightful elucidation of the association of epigenetic regulators with phagocytosis and supply important clues for therapeutic intervention in inflammatory and autoimmune diseases.

## Ethics Statement

This study was carried out in accordance with the recommendations of the Institutional Animal Care and Use Committee of the Zhejiang University Laboratory Animal Center. The protocol was approved by the Institutional Animal Care and Use Committee of the Zhejiang University Laboratory Animal Center.

## Author Contributions

LW and ZH designed the research. ZH, ZX, LFZ, YL, and FL performed the experiments. ZH, LNZ, and LW wrote the manuscript. LL, DW, JW, SH, XL, and WL provided expertise and advice. LW supervised the project.

## Conflict of Interest Statement

The authors declare that the research was conducted in the absence of any commercial or financial relationships that could be construed as a potential conflict of interest.

## References

[1] Hirayama D Iida T Nakase H. The phagocytic function of macrophage-enforcing innate immunity and tissue homeostasis. Int J Mol Sci (2018) 19(1):92. 10.3390/ijms19010092 29286292 PMC5796042

[2] Flannagan RS Jaumouille V Grinstein S. The cell biology of phagocytosis. Annu Rev Pathol (2012) 7:61–98. 10.1146/annurev-pathol-011811-132445 21910624

[3] Kapellos TS Iqbal AJ. Epigenetic control of macrophage polarisation and soluble mediator gene expression during inflammation. Mediators Inflamm (2016) 2016:6591703. 10.1155/2016/6591703 27143818 PMC4842078

[4] Alvarez-Errico D Vento-Tormo R Sieweke M Ballestar E. Epigenetic control of myeloid cell differentiation, identity and function. Nat Rev Immunol (2015) 15(1):7–17. 10.1038/nri3777 25534619

[5] Kouzarides T. Chromatin modifications and their function. Cell (2007) 128(4):693–705. 10.1016/j.cell.2007.02.005 17320507

[6] Li B Carey M Workman JL. The role of chromatin during transcription. Cell (2007) 128(4):707–19. 10.1016/j.cell.2007.01.015 17320508

[7] Zhou VW Goren A Bernstein BE. Charting histone modifications and the functional organization of mammalian genomes. Na Rev Genet (2011) 12(1):7–18. 10.1038/nrg2905 21116306

[8] Villagra A Cheng F Wang HW Suarez I Glozak M Maurin M The histone deacetylase HDAC11 regulates the expression of interleukin 10 and immune tolerance. Nat Immunol (2009) 10(1):92–100. 10.1038/ni.1673 19011628 PMC3925685

[9] Cao Q Rong S Repa JJ Clair RWS Parks JS Mishra N. Histone deacetylase 9 represses cholesterol efflux and alternatively activated macrophages in atherosclerosis development. Arterioscler Thromb Vasc Biol (2014) 34(9):1871–9. 10.1161/ATVBAHA.114.303393 25035344 PMC4217086

[10] Satoh T Takeuchi O Vandenbon A Yasuda K Tanaka Y Kumagai Y The Jmjd3-Irf4 axis regulates M2 macrophage polarization and host responses against helminth infection. Nat Immunol (2010) 11(10):936–44. 10.1038/ni.1920 20729857

[11] Austenaa L Barozzi I Chronowska A Termanini A Ostuni R Prosperini E The histone methyltransferase Wbp7 controls macrophage function through GPI glycolipid anchor synthesis. Immunity (2012) 36(4):572–85. 10.1016/j.immuni.2012.02.016 22483804

[12] Lee JH Skalnik DG. CpG-binding protein (CXXC finger protein 1) is a component of the mammalian Set1 histone H3-Lys4 methyltransferase complex, the analogue of the yeast Set1/COMPASS complex. J Biol Chem (2005) 280(50):41725–31. 10.1074/jbc.M508312200 16253997

[13] Clouaire T Webb S Skene P Illingworth R Kerr A Andrews R Cfp1 integrates both CpG content and gene activity for accurate H3K4me3 deposition in embryonic stem cells. Genes Dev (2012) 26(15):1714–28. 10.1101/gad.194209.112 22855832 PMC3418589

[14] Thomson JP Skene PJ Selfridge J Clouaire T Guy J Webb S CpG islands influence chromatin structure via the CpG-binding protein Cfp1. Nature (2010) 464(7291):1082–6. 10.1038/nature08924 20393567 PMC3730110

[15] Brown DA Di Cerbo V Feldmann A Ahn J Ito S Blackledge NP The SET1 complex selects actively transcribed target genes via multivalent interaction with CpG Island chromatin. Cell Rep (2017) 20(10):2313–27. 10.1016/j.celrep.2017.08.030 28877467 PMC5603731

[16] Clouaire T Webb S Bird A. Cfp1 is required for gene expression-dependent H3K4 trimethylation and H3K9 acetylation in embryonic stem cells. Genome Biol (2014) 15(9):451. 10.1186/s13059-014-0451-x 25201068 PMC4189735

[17] Cao W Guo J Wen X Miao L Lin F Xu G CXXC finger protein 1 is critical for T-cell intrathymic development through regulating H3K4 trimethylation. Nat Commun (2016) 7:11687. 10.1038/ncomms11687 27210293 PMC4879243

[18] Zhang W Wang X Xia X Liu X Suo S Guo J Klf10 inhibits IL-12p40 production in macrophage colony-stimulating factor-induced mouse bone marrow-derived macrophages. Eur J Immunol (2013) 43(1):258–69. 10.1002/eji.201242697 23065757 PMC3842096

[19] Lou J Li X Huang W Liang J Zheng M Xu T SNX10 promotes phagosome maturation in macrophages and protects mice against Listeria monocytogenes infection. Oncotarget (2017) 8(33):53935. 10.18632/oncotarget.19644 28903313 PMC5589552

[20] Geng J Sun X Wang P Zhang S Wang X Wu H Kinases Mst1 and Mst2 positively regulate phagocytic induction of reactive oxygen species and bactericidal activity. Nat Immunol (2015) 16(11):1142–52. 10.1038/ni.3268 26414765 PMC4618176

[21] Kim D Langmead B Salzberg SL. HISAT: a fast spliced aligner with low memory requirements. Nat Methods (2015) 12(4):357–60. 10.1038/nmeth.3317 25751142 PMC4655817

[22] Carlone DL Skalnik DG. CpG binding protein is crucial for early embryonic development. Mol Cell Biol (2001) 21(22):7601–6. 10.1128/MCB.21.22.7601-7606.2001 11604496 PMC99931

[23] Kristin T Chun BL Dobrota E Tate C Lee JH Khan S The epigenetic regulator CXXC FingerProtein 1 is essential for murine hematopoiesis. PLoS One (2014) 9(12):e113745. 10.1371/journal.pone.0113745 25470594 PMC4254612

[24] Hamon M Bierne H Cossart P. Listeria monocytogenes: a multifaceted model. Nat Rev Microbiol (2006) 4(6):423–34. 10.1038/nrmicro1413 16710323

[25] Huang da W Sherman BT Lempicki RA. Systematic and integrative analysis of large gene lists using DAVID bioinformatics resources. Nat Protoc (2009) 4(1):44–57. 10.1038/nprot.2008.211 19131956

[26] Nakamura K Funakoshi H Miyamoto K Tokunaga F Nakamura T. Molecular cloning and functional characterization of a human scavenger receptor with C-type lectin (SRCL), a novel member of a scavenger receptor family. Biochem Biophys Res Commun (2001) 280(4):1028–35. 10.1006/bbrc.2000.4210 11162630

[27] Nakamura K Funakoshi H Tokunaga F Nakamura T Molecular cloning of a mouse scavenger receptor with C-type lectin (SRCL)^1^, a novel member of the scavenger receptor family. Biochim Biophys Acta (2001) 1522(1):53–8. 10.1016/S0167-4781(01)00284-6 11718900

[28] Jang S Ohtani K Fukuoh A Yoshizaki T Fukuda M Motomura W Scavenger receptor collectin placenta 1 (CL-P1) predominantly mediates zymosan phagocytosis by human vascular endothelial cells. J Biol Chem (2009) 284(6):3956–65. 10.1074/jbc.M807477200 19073604

[29] Lambeth JD NOX enzymes and the biology of reactive oxygen. Nat Rev Immunol (2004) 4(3):181–9. 10.1038/nri1312 15039755

[30] Hercus TR Thomas D Guthridge MA Ekert PG King-Scott J Parker MW The granulocyte-macrophage colony-stimulating factor receptor: linking its structure to cell signaling and its role in disease. Blood (2009) 114(7):1289–98. 10.1182/blood-2008-12-164004 19436055 PMC2727416

[31] Becher B Tugues S Greter M. GM-CSF: from growth factor to central mediator of tissue inflammation. Immunity (2016) 45(5):963–73. 10.1016/j.immuni.2016.10.026 27851925

[32] Chen H Zhang P Voso M Hohaus S Gonzalez D Glass C Neutrophils and monocytes express high levels of PU. 1 (Spi-1) but not Spi-B. Blood (1995) 85(10):2918–28. 7742552

[33] Yoko Shibata P-YB Chroneos ZC Yoshida M Whitsett JA Trapnell BC. GM-CSF regulates alveolar macrophage differentiation and innate immunity in the lung through PU.1. Immunity (2001) 15(4):557–67. 10.1016/S1074-7613(01)00218-7 11672538

[34] Trapnell BC Whitsett JA. Gm-CSF regulates pulmonary surfactant homeostasis and alveolar macrophage-mediated innate host defense. Annu Rev Physiol (2002) 64:775–802. 10.1146/annurev.physiol.64.090601.113847 11826288

[35] Guilliams M De Kleer I Henri S Post S Vanhoutte L De Prijck S Alveolar macrophages develop from fetal monocytes that differentiate into long-lived cells in the first week of life via GM-CSF. J Exp Med (2013) 210(10):1977–92. 10.1084/jem.20131199 24043763 PMC3782041

[36] Suzuki T Arumugam P Sakagami T Lachmann N Chalk C Sallese A Pulmonary macrophage transplantation therapy. Nature (2014) 514(7523):450–4. 10.1038/nature13807 25274301 PMC4236859

[37] Schneider C Nobs SP Heer AK Kurrer M Klinke G van Rooijen N Alveolar macrophages are essential for protection from respiratory failure and associated morbidity following influenza virus infection. PLoS Pathog (2014) 10(4):e1004053. 10.1371/journal.ppat.1004053 24699679 PMC3974877

[38] Xia M Liu J Wu X Liu S Li G Han C Histone methyltransferase Ash1l suppresses interleukin-6 production and inflammatory autoimmune diseases by inducing the ubiquitin-editing enzyme A20. Immunity (2013) 39(3):470–81. 10.1016/j.immuni.2013.08.016 24012418

[39] Yu C Fan X Sha Q-Q Wang H-H Li B-T Dai X-X CFP1 regulates histone H3K4 trimethylation and developmental potential in mouse oocytes. Cell Rep (2017) 20(5):1161–72. 10.1016/j.celrep.2017.07.011 28768200

[40] Carson WF Cavassani KA Soares EM Hirai S Kittan NA Schaller MA The STAT4/MLL1 epigenetic axis regulates the antimicrobial functions of murine macrophages. J Immunol (2017) 199(5):1865–74. 10.4049/jimmunol.1601272 28733487 PMC5568492

[41] Berclaz PY Shibata Y Whitsett JA Trapnell BC. GM-CSF, via PU.1, regulates alveolar macrophage Fcgamma R-mediated phagocytosis and the IL-18/IFN-gamma -mediated molecular connection between innate and adaptive immunity in the lung. Blood (2002) 100(12):4193–200. 10.1182/blood-2002-04-1102 12393686

[42] Suzuki T Sakagami T Rubin BK Nogee LM Wood RE Zimmerman SL Familial pulmonary alveolar proteinosis caused by mutations in CSF2RA. J Exp Med (2008) 205(12):2703–10. 10.1084/jem.20080990 18955570 PMC2585845

