# Supplementary material for: Cxxc Finger Protein 1 Positively Regulates GM-CSF-Derived Macrophage Phagocytosis Through Csf2rα-Mediated Signaling
Source: Front Immunol. 2018 Aug 14;9:1885. doi: 10.3389/fimmu.2018.01885 (PMC6102347; doi:10.3389/fimmu.2018.01885)
Supplement: Supplementary file 1 [file Data_Sheet_1.PDF]

Supplementary Material

Supplemental Figure 1 | CFP1-deficient mice had few defects in macrophage development. **(A, B)** The mRNA and protein levels of CFP1 in wild-type (black rectangle) and CFP1-deficient macrophages (white rectangle) macrophages. **(C, D, E, F)** Flow cytometry analysing F4/80$^+$CD11b$^+$ macrophages and CD11b$^+$Ly6C$^+$ monocytes in the bone marrow and spleen of wild-type (black rectangle) and CFP1-deficient macrophages (white rectangle) mice. **(G)** FACS analysis of F4/80, CD11b, MHC II, CD80 and CD86 in wild-type and CFP1-deficient macrophages. The mean and SD of three independent experiments are shown.

Supplemental Figure 2 | CFP1 had few effects on proinflammatory cytokine production after stimulation with LTA or LPS. **(A, B)** The mRNA level of TNF-α, IL-6, iNOS and IL-12p40 in wild-type (black rectangle) and CFP1-deficient macrophages (white rectangle) after simulation with LTA (2 μg/ml) **(A)** and LPS (100 ng/ml) **(B)** The levels at 0, 3, and 6 h, normalized to Gapdh mRNA. **(C)** Enzyme-linked immunosorbent assay (ELISA) to determine TNF-α, IL-6, iNOS and IL-12p40 levels in the supernatants of wild-type (black rectangle) and CFP1-deficient macrophages (white rectangle) macrophages stimulated with LTA (2 μg/ml) or LPS (100 ng/ml) 12 h after stimulation. The mean and SD of three independent experiments are shown. **P < 0.01 and ***P < 0.001 indicate significant differences between groups as determined by Student's t-test.

Supplemental Figure 3 | CFP1-deficient mice had more liver and spleen damage following *L. monocytogenes* infection than did wild-type mice. **(A)** The number of liver abscess in wild-type (white circle) and CFP1-deficient macrophages (white square) was quantitated from H&E images and is presented as the mean liver abscess number and SD. **(B)** The number of intact white pulp in the spleen of wild-type (white circle) and CFP1-deficient macrophages (white square) was quantitated from the H&E images and is presented as the mean intact white pulp number and SD. Each symbol represents an individual mouse; the small horizontal lines indicate the mean **(A, B)**. *P < 0.05 and ***P < 0.001 indicate significant differences between groups as determined by

Student's t-test.

Supplemental Materials and Methods

RNA Isolation and Real-Time PCR (Q-PCR)

Total RNA was extracted using RNAiso Plus (TaKaRa). The cDNA was synthesized from total RNA using a PrimeScript™ RT-PCR kit (TaKaRa). Real-time PCR was performed using a 96-well CFX-96 detection system (Bio-Rad Laboratories) with SYBR Premix Ex Taq™ (TaKaRa, DRR041A). The corresponding primers are listed in Supplementary Table 2.

Cytokine Measurement

Cell supernatants were collected at the appropriate time points after stimulation with LTA (InvivoGen, 2 μg/ml) or LPS (Sigma, 100 ng/ml). The concentrations of mouse TNFα (8-1000pg/ml), IL-6 (4-500pg/ml), or IL-12p40 (2-300pg/ml) were measured with ELISA kits (eBioscience) according to the manufacturers' instructions.

Detection of NO in Culture Supernatants

Cell supernatants were analysed for NO by the Griess reaction (S0021, Beyotime Biotechnology).

Western Blotting and Antibodies

Bone marrow-derived macrophages were lysed in complete lysis-M buffer (Roche; cat. no. 04719956001). Total protein was subjected to SDS–PAGE and immunoblotting and was transferred onto nitrocellulose membranes for incubation with antibodies.
Anti-Csf2rα (0804-8,1:500 dilution) was from Huabio. Anti-CFP1 (ab56035, 1:500 dilution) was from Abcam. Anti-pSTAT5 and anti-STAT5 were from Cell Signaling Technology. Anti-COLEC12, anti-NCF1 and anti-NCF2 were from Sangon Biotech.

Plasmid Construction

Recombinant vectors encoding murine Csf2rα, PU.1, and CFP1 and the full-length

constructs were constructed by PCR-based amplification and subcloning into the pMX-IRES-GFP vector.

Data availability

The RNA-seq data sets were deposited in Sequence Read Archive (SRA), with an accession number of SRP131829, are available via the repository's data access request procedures.
