# Supplementary figures and images for: Cxxc Finger Protein 1 Positively Regulates GM-CSF-Derived Macrophage Phagocytosis Through Csf2rα-Mediated Signaling
Source: Front Immunol. 2018 Aug 14;9:1885. doi: 10.3389/fimmu.2018.01885 (PMC6102347; doi:10.3389/fimmu.2018.01885)

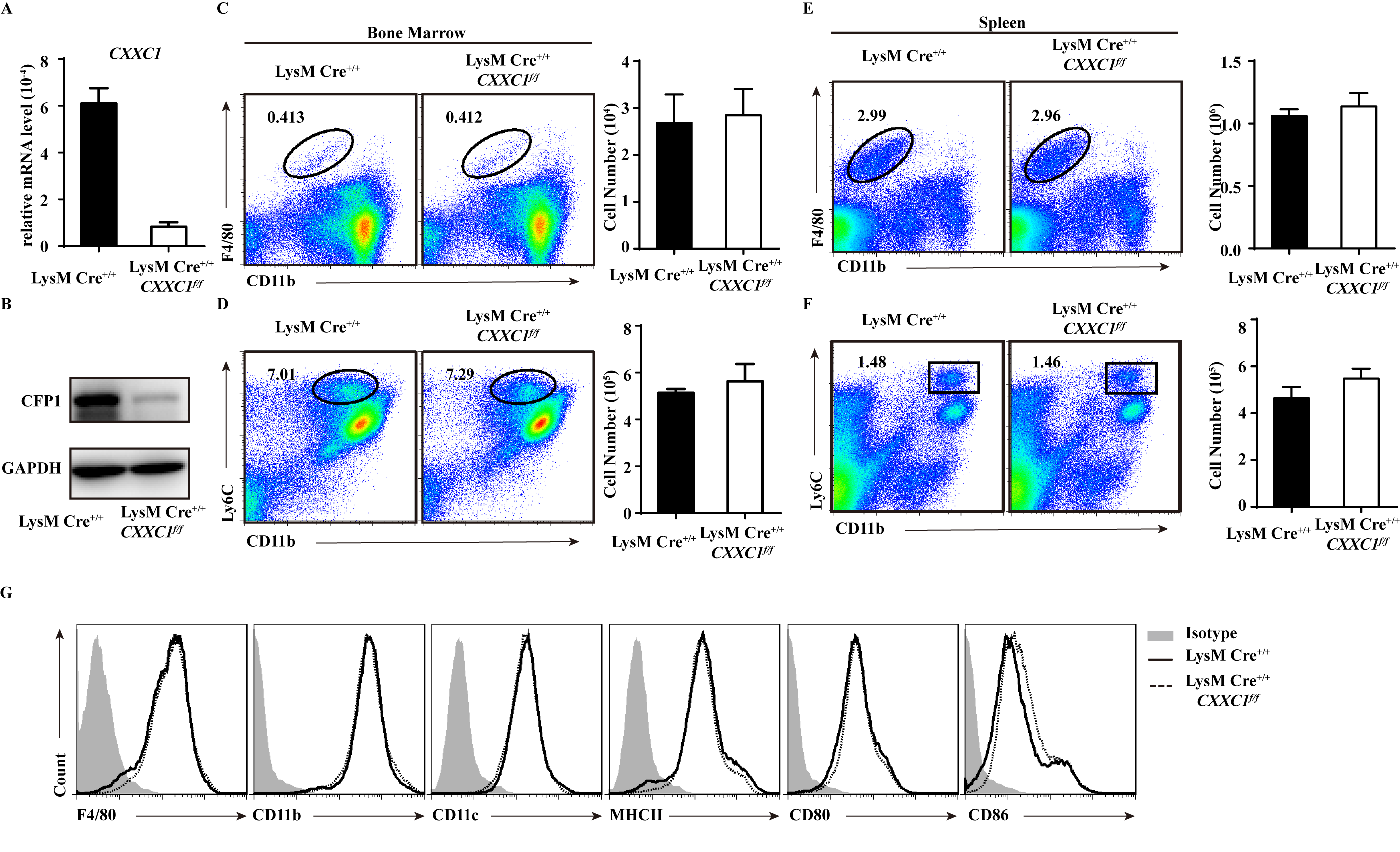

Supplement: Supplementary file 2 [file Image_1.tif]

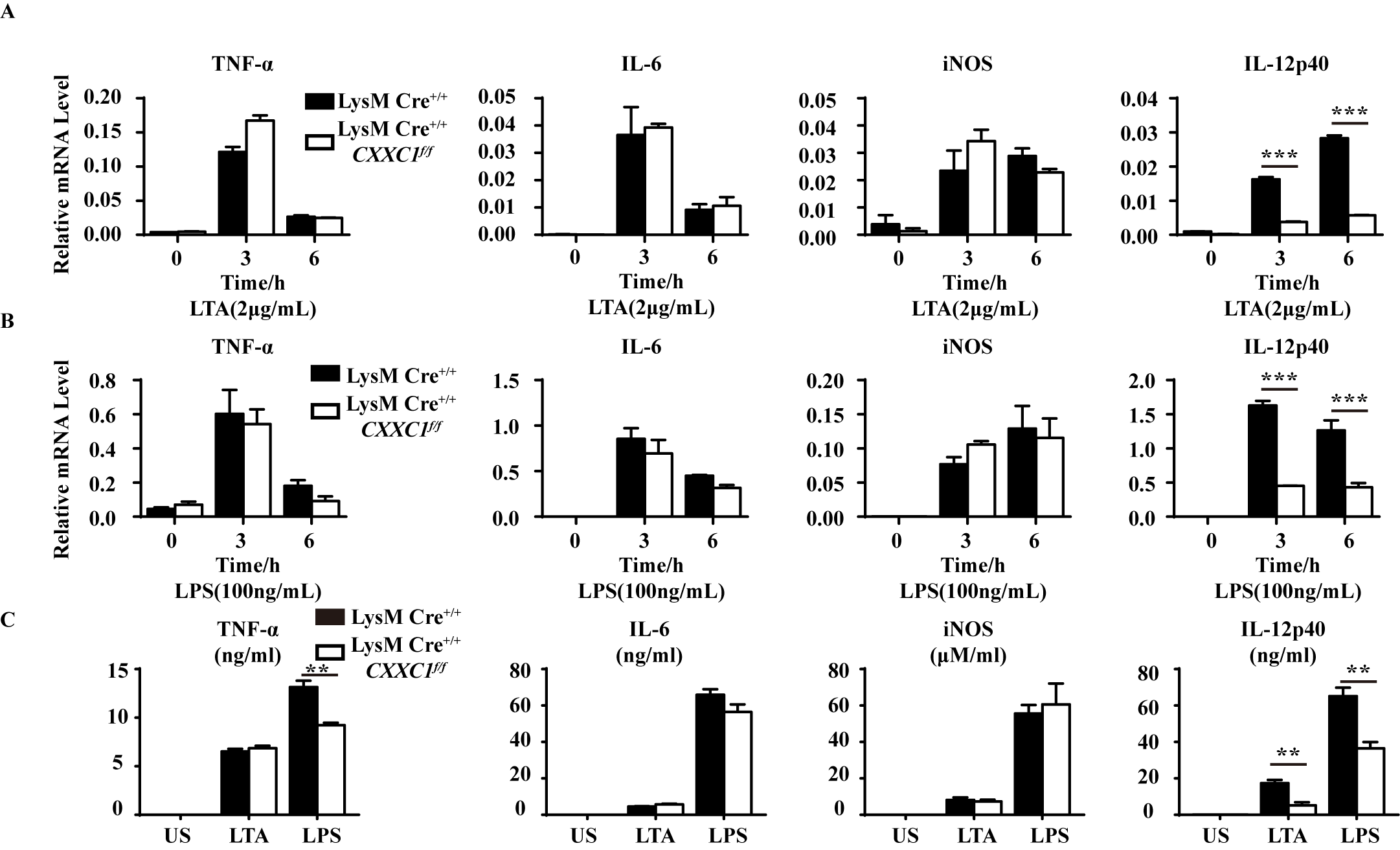

Supplement: Supplementary file 3 [file Image_2.tif]

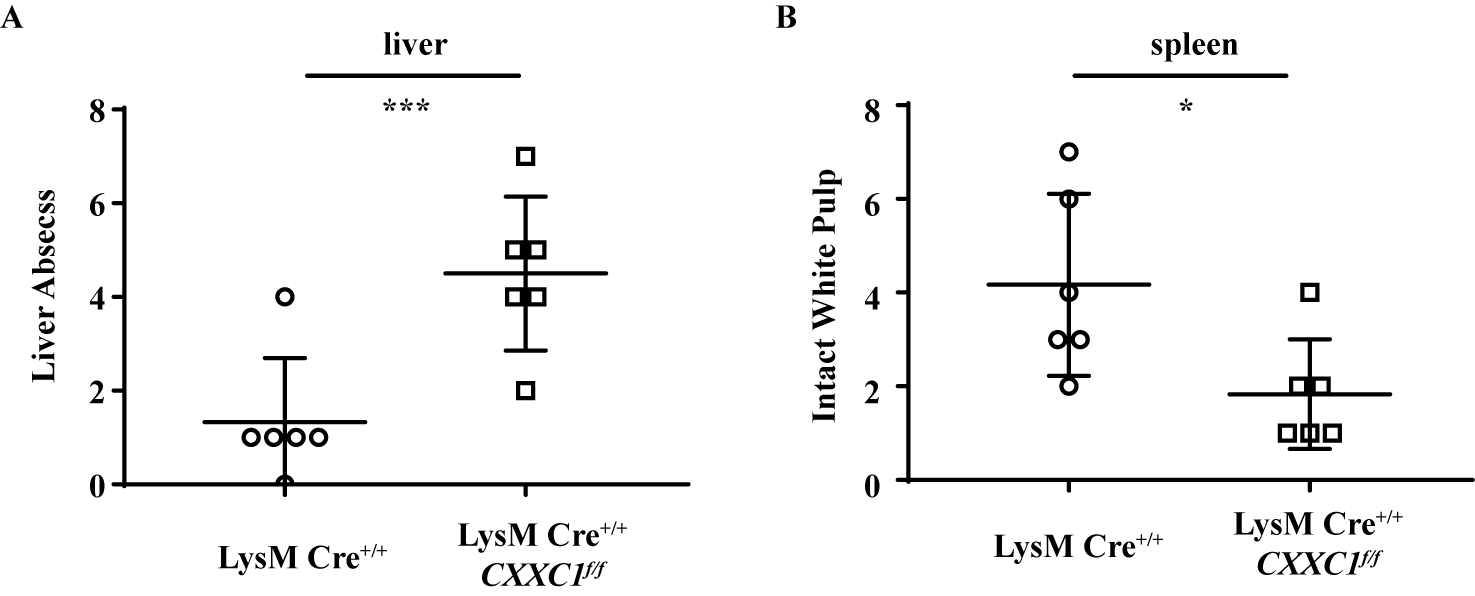

Supplement: Supplementary file 4 [file Image_3.tif]
